# Data mining approaches to pneumothorax detection: Integrating mask-RCNN and medical transfer learning techniques

**DOI:** 10.1016/j.mex.2024.102692

**Published:** 2024-04-03

**Authors:** Shwetambari Chiwhane, Lalit Shrotriya, Amol Dhumane, Sonali Kothari, Deepak Dharrao, Pooja Bagane

**Affiliations:** Symbiosis Institute of Technology – Pune Campus, Symbiosis International (Deemed University), Pune, India

**Keywords:** Pneumothorax detection, Region based convolutional neural network (R-CNN), Area under the ROC Curve (AUC), Receiver operating characteristic curve (ROC), Data Mining Approaches to Pneumothorax Detection

## Abstract

With the medical condition of pneumothorax, also known as collapsed lung, air builds up in the pleural cavity and causes the lung to collapse. It is a critical disorder that needs to be identified and treated right as it can cause breathing difficulties, low blood oxygen levels, and, in extreme circumstances, death. Chest X-rays are frequently used to diagnose pneumothorax. Using the Mask R-CNN model and medical transfer learning, the proposed work offers

• A novel method for pneumothorax segmentation from chest X-rays.
• A method that takes advantage of the Mask R-CNN architecture's for object recognition and segmentation.
• A modified model to address the issue of segmenting pneumothoraxes and then polish it using a sizable dataset of chest X-rays.

The proposed method is tested against other pneumothorax segmentation techniques using a dataset of ‘chest X-rays’ with ‘pneumothorax annotations. The test findings demonstrate that proposed method outperforms other cutting-edge techniques in terms of segmentation accuracy and speed. The proposed method could lead to better patient outcomes by increasing the precision and effectiveness of pneumothorax diagnosis and therapy. Proposed method also benefits other medical imaging activities by using the medical transfer learning approaches which increases the precision of computer-aided diagnosis and treatment planning.

## Graphical abstract

Attached

**Figure:**
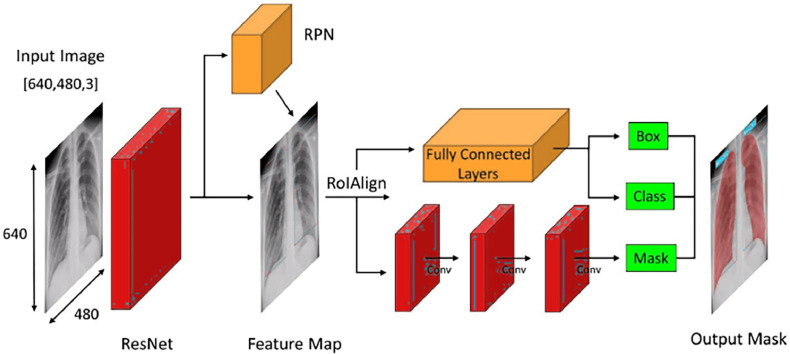

Specifications table

**Table:** 

Subject area:	Computer Science
More specific subject area:	Data Mining and Deep Learning
Name of your method:	Data Mining Approaches to Pneumothorax Detection
Name and reference of original method:	NA
Resource availability:	NA

**Method details**

## Introduction

Pneumothorax, which is also referred to as collapsed lung, is a medical ailment where air gathers in the pleural cavity resulting in the collapse of the lung. This is a critical condition that needs to be diagnosed and treated quickly as it can cause respiratory difficulties, low levels of oxygen in the blood, and in severe cases, even death. Chest X-rays are commonly utilized for diagnosing pneumothorax because they offer a cost-effective and non-invasive means of observing the lung and identifying the presence of air in the pleural cavity.

Although chest X-rays are commonly used to diagnose pneumothorax, identifying the condition on an X-ray can be a time-consuming and challenging task that demands a high level of skill and experience. This is because the signs of pneumothorax can be very subtle and not easily discernible to the human eye. The need for more accurate pneumothorax diagnosis has prompted the development of computer-aided diagnostic (CAD) systems to aid radiologists in reading chest X-rays. However, conventional computer vision techniques such as thresholding and contour detection have limitations because they can produce false negatives or false positives due to their sensitivity to variations in X-ray intensity.

To address this issue, deep learning models have recently emerged as a promising tool for medical image analysis, including the segmentation of pneumothorax. Among these models, Mask R- CNN has demonstrated exceptional performance in detecting and segmenting objects, and has the potential to be a powerful tool for pneumothorax segmentation. However, the large size and high computational demands of deep learning models pose challenges for training on small medical image datasets, leading to overfitting and diminished performance.

To overcome this challenge, a popular deep learning technique known as transfer learning can be used to fine-tune a pre-trained model on a smaller dataset for a target task. This approach is advantageous because it allows the fine-tuned model to benefit from the knowledge gained from the pre- trained model, which can enhance its performance on the target task even when the training data is limited.

In this research paper, a Mask R-CNN model for pneumothorax segmentation in chest X-rays that uses transfer learning is proposed. The objective is to evaluate the performance of the fine-tuned Mask R-CNN model for pneumothorax segmentation and compare it with traditional computer vision techniques and other advanced deep learning models. The results of this study provide valuable insights into the potential of Mask R-CNN and transfer learning for pneumothorax segmentation and may help advance the development of AI-based CAD systems for pneumothorax diagnosis.

The subsequent sections of the paper contain a comprehensive description of the Mask R-CNN model, transfer learning, and the experimental setup used in this study. The proposed work also report and analyze the outcomes of proposed experiments and discuss the implications of proposed findings for future research in the field of medical image analysis.

## Literature review

The identification and segmentation of pneumothorax in chest X-rays is a topic of significant interest in medical image analysis research. This section provides an overview of studies related to pneumothorax detection and segmentation, deep learning models applied to medical image analysis, and the use of transfer learning in medical imaging.

In their research [1], the authors suggest a deep learning model for detecting pneumothorax that can be used to assess the model's generalizability on datasets collected from various external institutions. They also examined patient-related and acquisition-related factors that may influence the performance and results of the model. The researchers trained the proposed model on combined data from two open-source datasets that contained radiographs, namely ChestX-ray14 and CheXpert. The model was tested on six different datasets from independent institutions that contained retrospective data in a case-control manner. To evaluate the performance of the model on each dataset, the researchers used the area under the ROC (Receiving Operating Characteristic) curve and analyzed specificity and sensitivity, using two radiologists as a reference standard. Additionally, the researchers analyzed various factors that may influence the performance and results of the deep learning model. For the results, the respective areas under the curves for the 6 datasets that were used are 0.91, 0.97, 0.91, 0.98, 0.97 and 0.92 (for the datasets labelled A-F respectively) and for internal dataset the area under curve was 0.93. While comparing the model's performance on pneumothoraxes that were large and small it was found out the AUC for large pneumothoraxes was more (0.97) as compared to AUC for small pneumothoraxes which was 0.88. The model performed approximately the same on data with radiographs containing or not containing the chest tube where the AUCs were 0.95 and 0.94 respectively. In conclusion, their model had well generalizability on several externally acquired datasets [1].

The authors of [2], utilized transfer learning in their deep learning model, which used a Residual Neural Network as a base, and consisted of two stages for identifying pneumothorax: LFL (Local Feature Learning) and GMIL (global multi-instance learning). To enable learning of discriminative features, non-lesion cell regions were removed during pre-processing. Two datasets were used for model validation - one containing 27,955 front view X-ray images from a private dataset, and another public dataset from the National Institute of Health containing approximately 112,120 frontal view X-ray images. To evaluate model performance, metrics such as accuracy, precision, recall, specificity, F1-score, ROC, and AUC ROC were used. Mean and standard deviation of these metrics from 5-fold cross-validation results were used to assess the model's performance. The results showed an accuracy of 94.4% (mean) with a standard deviation of 0.7%, AUC of 97.3% (mean) with a standard deviation of 0.5%, precision of 94.2% (mean) with a standard deviation of 0.3%, recall of 94.6% (mean) with a standard deviation of 1.5%, specificity of 94.2% (mean) with a standard deviation of 0.4%, and F1-Score of 94.4% (mean) with a standard deviation of 0.7% on the public dataset from the National Institutes of Health. In conclusion, the study demonstrated that transfer learning can achieve state-of-the-art performance for detecting pneumothorax from X-ray images [14].

The authors of [3], used a CNN model to detect pneumothorax on chest CT. The model contained 8 layers having a constant size of 36 by 36 pixels of 2D image patches. For training a dataset containing 80 CT images was used that had 50 images labelled as with and the rest 30 labelled as without pneumothorax. Classification of the image patches was done by the probability that they represented pneumothorax as 3D heat maps were subsequently generated. The heat maps incorporated information about the size of the pneumothorax area, the location of the region relative to the lung boundary, and a shape descriptor that utilized the region's anisotropy. For classification of the images at the end, SVM was used as probabilities needed to be generated for each individual image patch. For performance assessment, another dataset containing 200 CT images of the chest was used that had 75% images with and the rest 25% without pneumothorax. Accuracy, sensitivity, and specificity were analyzed. For images with pneumothorax, all of them were classified correctly yielding a sensitivity of 100%, and around 33 images without pneumothorax out of 40 were detected as true negatives, resulting in around 82.5% specificity. Emphysema and other artifacts in the test data images were the main driving factor for false positive results. To summarize, the model that was suggested has demonstrated a very high level of accuracy in detecting pneumothorax from CT images of the chest.

To detect moderate or large pneumothorax that could be potentially life-threatening, the authors of [4], employed deep convolutional networks trained on a large dataset of chest X- ray images that were manually annotated by radiologists [13]. Specifically, the radiologists annotated approximately 13,292 frontal view X-rays of the chest, categorizing images with large or moderate pneumothorax as positive and those with only a little trace as negative. Images containing small amounts of pneumothorax that could not be clearly categorized were excluded from the training data. Multiple models were tested on this dataset with an internal validation dataset containing 1993 images, and 2 best performing models which were finally evaluated using a test dataset that was held out internally. The metrics used were AUC ROC, sensitivity, specificity, and positive predictive value. Finally, an external set was used for determining performance, initially with a small subset and then with the full set that included small extent of pneumothorax labelled has positive. The model with high sensitivity achieved a test subset performance of 84% for sensitivity, 90% for specificity, 94% for AUC ROC, and a PPV of 0.45, when evaluated on data that did not include images with small pneumothorax. The other model with high specificity resulted in sensitivity of around 80%, specificity of 97%, and AUC ROC of 96%, along with PPV of 0.71. When testing with the full set that included the images with small pneumothorax images, it showed reduced performance, and even further decline was seen on using external NIH dataset.

The authors of [5], used a dataset of 19,237 CXR images that were manually labelled, named CANDID-PTX. The models were used for classification of images of pneumothorax as well as for segmentation of images. AUC ROC, sensitivity, specificity was used as metrics for evaluating the classification model whereas metrics such as mean dice and true positive dice coefficients were used for evaluating segmentation performance. And based on results, the model with best performance was selected for implementation of triage simulation. As a result, for the best performing model the metrics were 0.94 for AUC ROC, 0.93 for sensitivity and 0.95 for specificity. The most effective segmentation model achieved a positive dice coefficient of 0.69. In the triage simulation, the reporting delays for chest X-rays containing pneumothorax were reduced from an average of 9.8 days with a standard deviation of

2 days to an average of 1.0 day with a standard deviation of 0.5 days. The classification model demonstrated a sensitivity of 0.95 and specificity of 0.95. In the end, when interpretability was analyzed, it was seen that models made use of logic that was understandable for radiologists and produced very less bias or confounding while making predictions.

There have been several studies proposing computer-aided diagnostic (CAD) systems for detecting and segmenting pneumothorax in chest X-rays. One such study was done by Kim et al. [6], who developed a CAD system that uses a combination of morphological operations and thresholding to detect and segment pneumothorax in chest X-rays. Another study was conducted by Zhang et al. [7], who proposed a CAD system that utilized a deep convolutional neural network (DCNN) for pneumothorax segmentation. The DCNN was trained on a dataset of chest X-rays and achieved a high level of accuracy in detecting pneumothorax.

Deep learning models, particularly those based on convolutional neural networks (CNNs), have achieved significant success in various medical image analysis tasks, including object detection and segmentation. One such model is the region-based CNN (R-CNN) proposed by Girshick et al. [8], which utilizes a combination of CNN features and region proposals for object detection in images.

Razakarivony et al. [9], provides a comprehensive review of transfer learning in medical imaging. The authors survey various transfer learning approaches and their applications in medical imaging tasks, including image classification, segmentation, and diagnosis. The authors also discuss the challenges and limitations of transfer learning in medical imaging and provide insights for future work.

Wang, et al. [10], presents the ChestX-ray8 dataset, a large and diverse dataset of chest X-rays annotated with eight common thorax diseases. They also introduced the ChestX-ray8 dataset, which is a collection of over 100,000 chest X-rays that are annotated with eight different thoracic diseases. The dataset is intended for use in research on weakly-supervised classification and localization of these diseases. The authors also offer standards for identifying and locating thorax diseases through weakly-supervised classification and localization using the ChestX-ray8 dataset.

Seglar and Montesa [11], proposed a technique based on deep learning to automatically detect pneumothorax in chest X-rays. Their approach involved training a convolutional neural network (CNN) to classify chest X-rays as positive or negative for pneumothorax and evaluating the method's performance on a dataset of chest X-rays. The study found that the method achieved high accuracy in detecting pneumothorax.

## Methodology

To collect data, the researchers obtained images from a Kaggle dataset called SIIM ACR Pneumothorax Segmentation Data, which contained three folders (stage 2 images, dicom test, and dicom_train) with a total of 5 GB of images. The researchers also used SÍM-ACR Pneumothorax Segmentation and Mask- RCNN and COCO transfer learning LB:0.155 as additional sources of data.

• Dataset Distribution Balancing
• Image Augmentation
• Multi-Stage Training for Transfer Learning, and Model Weights Analysis
• Multi-Mask Prediction

The methodology followed resembles the following sequence:

1. Installing Matterport's Mask-RCNN
2. Setting up the Mask-RCNN
3. Examining the annotation data
4. Creating and preparing the training dataset
5. Image augmentation
6. Training the model
7. Validation process
8. Analysing the model weights

To segment pneumothorax in chest X-rays, a method is proposed that uses transfer learning by importing pre-trained weights that were originally used for pneumonia. The method has two stages, first training a Mask R-CNN model for pneumothorax segmentation, and then calibrating the pre-trained model on a smaller dataset of chest X-rays that have annotated pneumothorax.

The process for training and fine-tuning the Mask R-CNN model for pneumothorax segmentation in chest X-rays is composed of the following steps:

**Data collection:** A dataset of chest X-rays with annotated pneumothorax is collected. The annotations provide information about the location and size of the pneumothorax in each image.

**Data pre- processing:** The collected chest X-rays are standardized to a uniform size and normalized to zero mean and unit variance. The annotated pneumothorax regions are transformed into binary masks.

**Fig. 1:**
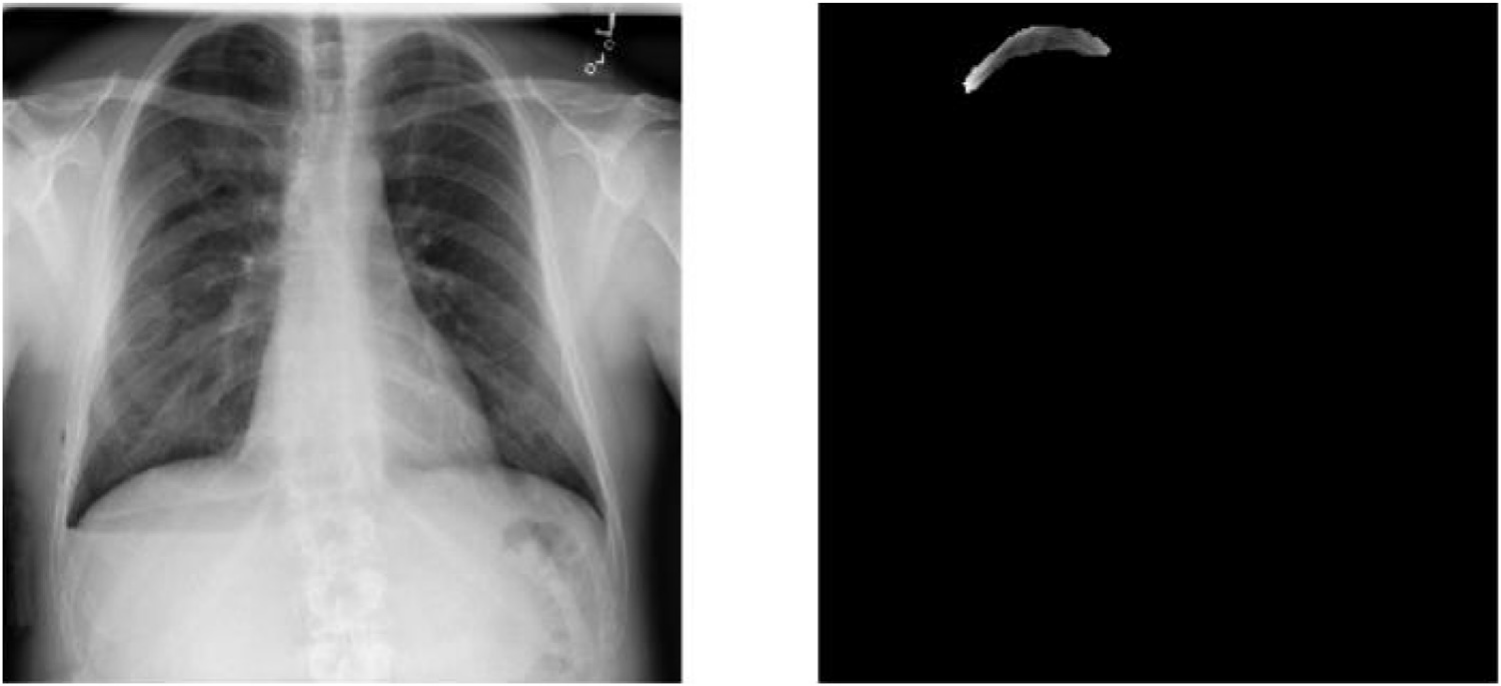
Dataset image for chest X ray.

**Model training:** The pre-processed chest X-rays and annotations are used to train the Mask R-CNN model using supervised learning. The model is trained to predict the location and size of the pneumothorax in each image and to segment the pneumothorax region in the image. The training process uses a standard optimization algorithm, such as stochastic gradient descent (SGD) or Adam, and a cross-entropy loss function. The model is trained for 13 epochs with a learning rate of 0.0006, which is doubled for Mask RCNN heads to expedite learning.

Fine-tuning the pre-trained Mask R-CNN model:

**Fine-tuning the pre-trained Mask R-CNN model:** A smaller dataset of chest X-rays with annotated pneumothorax is collected to fine-tune the pre-trained model [12]. The chest X-rays are pre-processed in the same way as the training data. The pre-trained Mask R-CNN model is fine-tuned on the smaller dataset of chest X-rays with annotated pneumothorax. The fine-tuning process updates the model parameters to better fit the target task of pneumothorax segmentation. The fine-tuning process uses a standard optimization algorithm and a cross-entropy loss function.

**Evaluation of the Mask R-CNN model:** The performance of the trained and fine-tuned Mask R-CNN model is evaluated on a held-out test dataset of chest X-rays with annotated pneumothorax. The evaluation metrics include the mean intersection over union (IoU) and the accuracy of the pneumothorax segmentation. The mean IoU measures the overlap between the predicted and ground-truth pneumothorax masks, while the accuracy measures the percentage of correctly predicted pixels in the pneumothorax region [12], [13], [14], [15].

Overall, the proposed method for pneumothorax segmentation using Mask R-CNN and medical transfer learning is a promising approach for accurate and efficient pneumothorax segmentation in chest X-rays. The method takes advantage of the power of deep learning models and transfer learning to achieve high performance on the target task.

To segment pneumothoraxes in chest X-ray images, the Mask R-CNN architecture can be divided into two main components.

(1) Firstly, a convolutional neural network (CNN) is utilized to extract information from the input image. This CNN can either be trained from scratch on the chest X-ray dataset or pre-trained on a large dataset such as ImageNet.
(2) Secondly, the Mask R-CNN head, which consists of three branches, is fed the output of the CNN.
  (a) The first branch predicts the bounding box for each object in the image,
  (b) The second branch predicts the class of each object (either pneumothorax or not pneumothorax), and
  (c) The third branch predicts a binary mask for each object in the image that shows the pixels belonging to the object (in this case, pneumothorax).

All three branches share the feature maps from the CNN, which enables them to learn a condensed representation of the input image while still making complex predictions. The final output of the Mask R- CNN is a set of bounding boxes, class predictions, and binary masks for each object in the image. Overall, the Mask R-CNN architecture offers a promising approach to accurately and efficiently segmenting pneumothoraxes in chest X-ray images.

This paper provides work a sophisticated image augmentation pipeline that has been carefully designed to improve the variety and resilience of datasets used in machine learning models for image analysis. The pipeline incorporates a series of geometric transformations, adjustments in brightness and contrast, and modifications in clarity, such as blurring and sharpening, in order to promote spatial diversity, adaptation to varying lighting conditions, and resilience against overfitting. Additionally, it is advisable to include denoising, enhancing, smoothing, and filtering methodologies in order to effectively mitigate real-world defects, enhance the visibility of features, diminish sharpness and pixelation, and accentuate the structural components of images. These extensive augmentation procedures greatly enhance the dataset, strengthening the ability of models to generalize and representing a considerable progress in the construction of robust and adaptable image analysis models. By employing this methodology, paper provide a novel standard for developing machine learning models that exhibit strong adaptability to diverse imaging scenarios, therefore advancing the frontiers of image analysis and machine learning.

**Fig. 2:**
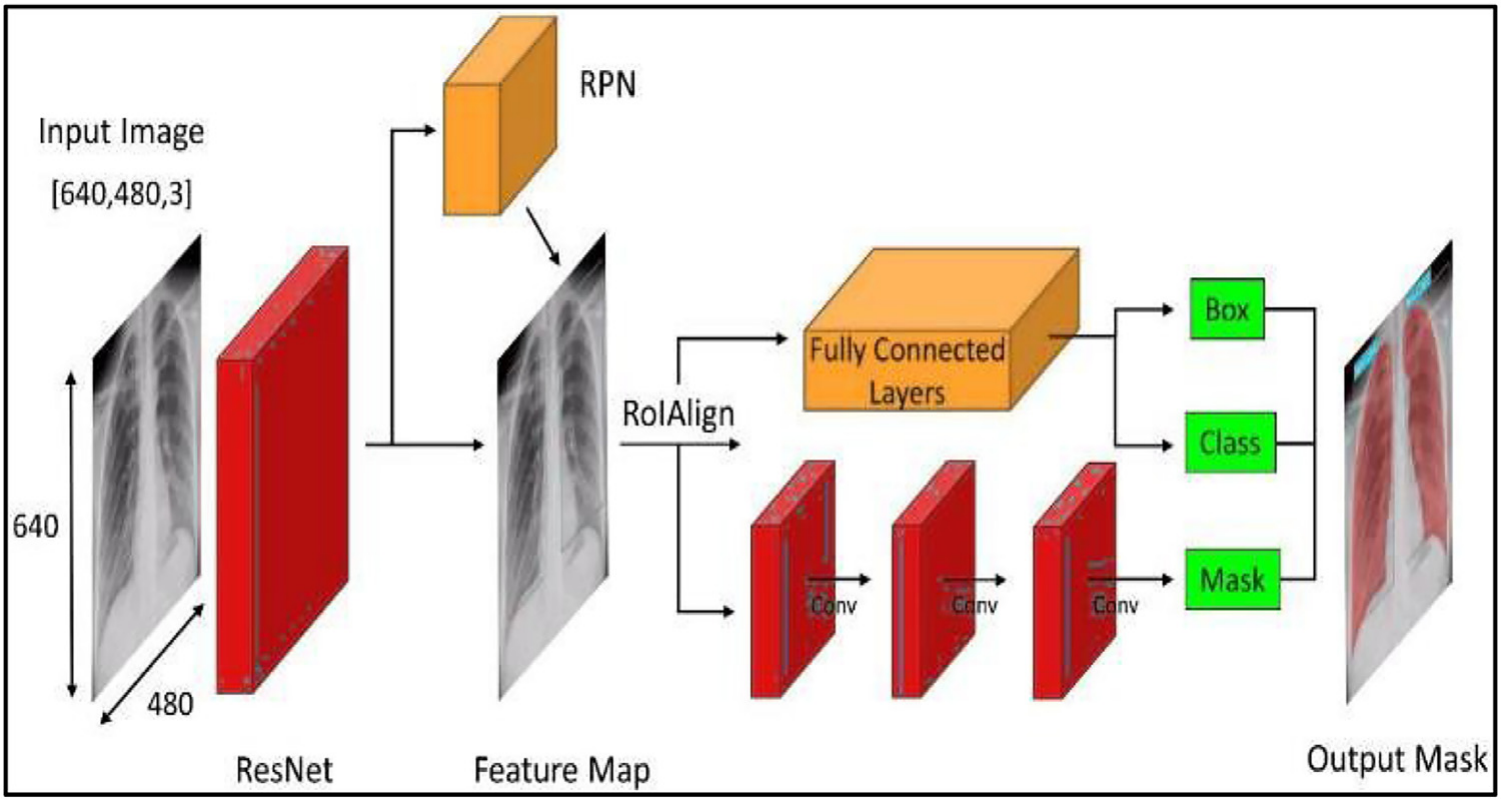
Architecture diagram of the model.

**Fig. 3:**
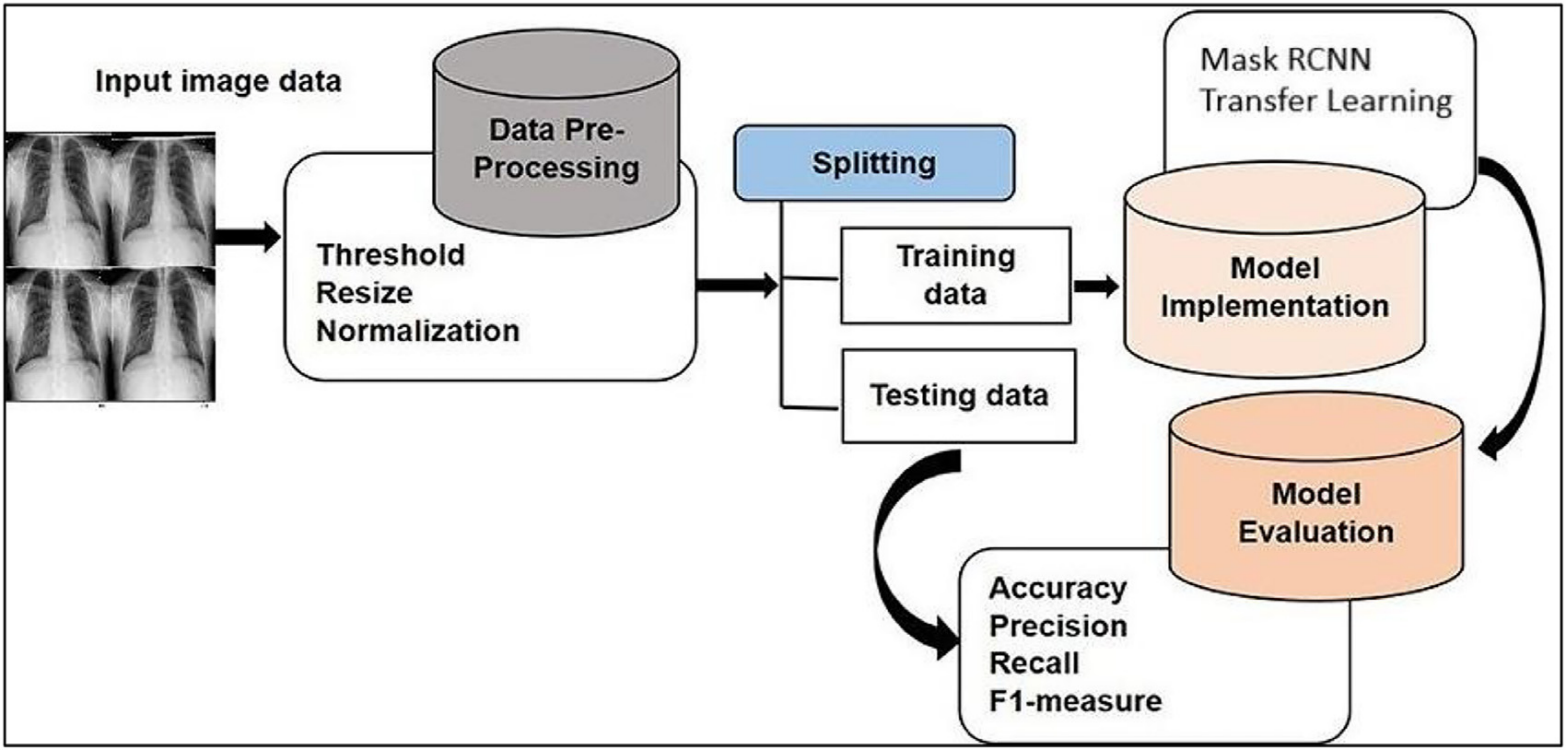
Workflow diagram for complete process of model architecture based on RCNN for Pneumothorax detection.

**Fig. 4:**
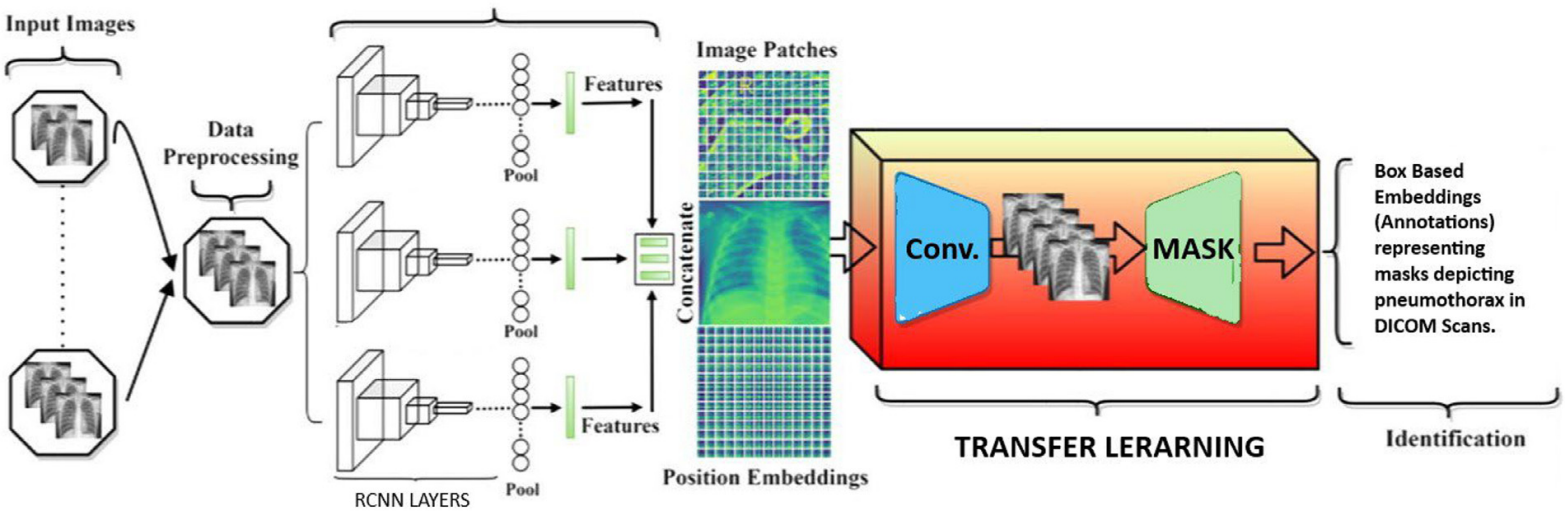
Algorithmic flowchart for complete process.

## Results and discussion

The model was correctly able to detect the presence of pneumothorax with its intensity in the CT images of chest. Shown below are some of the results that the model was able to achieve:

Following metrics were usewd to analyze the model's performance on train as well as validation data:

(1) Loss
(2) RPN Class Loss
(3) RPN Bbox Loss
(4) MRCNN Class Loss
(5) MRCNN Bbox Loss
(6) MRCNN Mask Loss

The values of mean median and mode for above metrics are displayed in the table below.

**Table 1:** Evaluation metrics values.

S. No.	Particulars	Minimum	Mean	Median	Variance
1	Loss	2.810919	3.414196923	3.309233	0.323487429
	Validation Loss	3.092739	3.401242	3.366223	0.079079279
2	RPN Class loss	0.265538	0.407395846	0.363212	0.027413468
	Validation RPN Class loss	0.327225	0.393248846	0.374058	0.004324203
3	RPN Bbox loss	0.278526	0.345426308	0.337207	0.004358125
	Validation BBox loss	0.309644	0.327674769	0.317186	0.000481754
4	MRCNN Class loss	1.174143	1.454201385	1.416989	0.044684
	Validation MRCNN Class loss	1.289619	1.478388	1.536243	0.017536
5	MRCNN Bbox loss	0.350579	0.395869	0.390063	0.00169
	Validation MRCNN Bbox loss	0.353601	0.39388	0.397364	0.000468
6	MRCNN Mask loss	0.720839	0.81121	0.775317	0.009526
	Validation MRCNN Mask loss	0.728628	0.807956	0.783314	0.004587

**Fig. 5:**
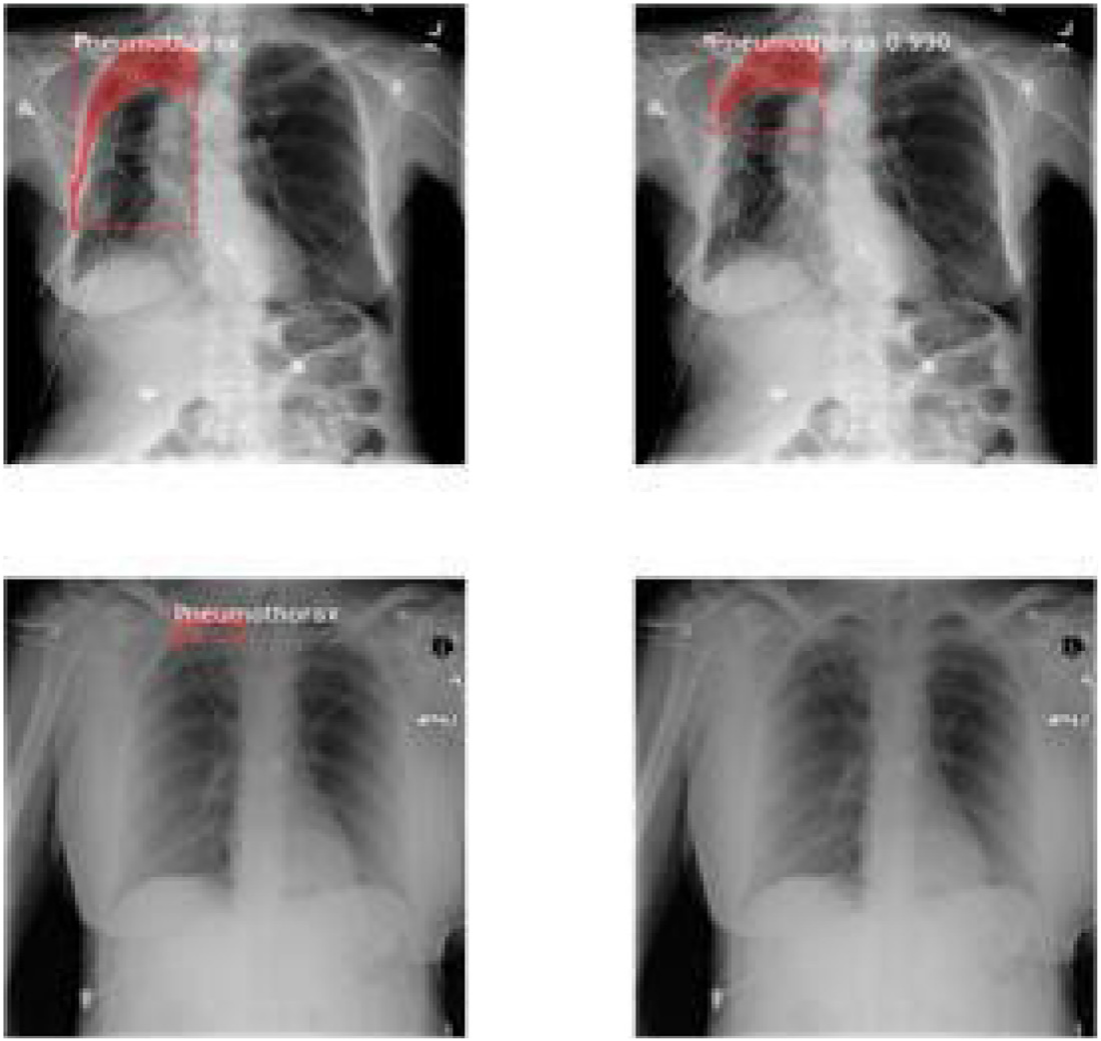
Results of the prediction.

Plots for each of the above metrics are shown below.

**Fig. 6:**
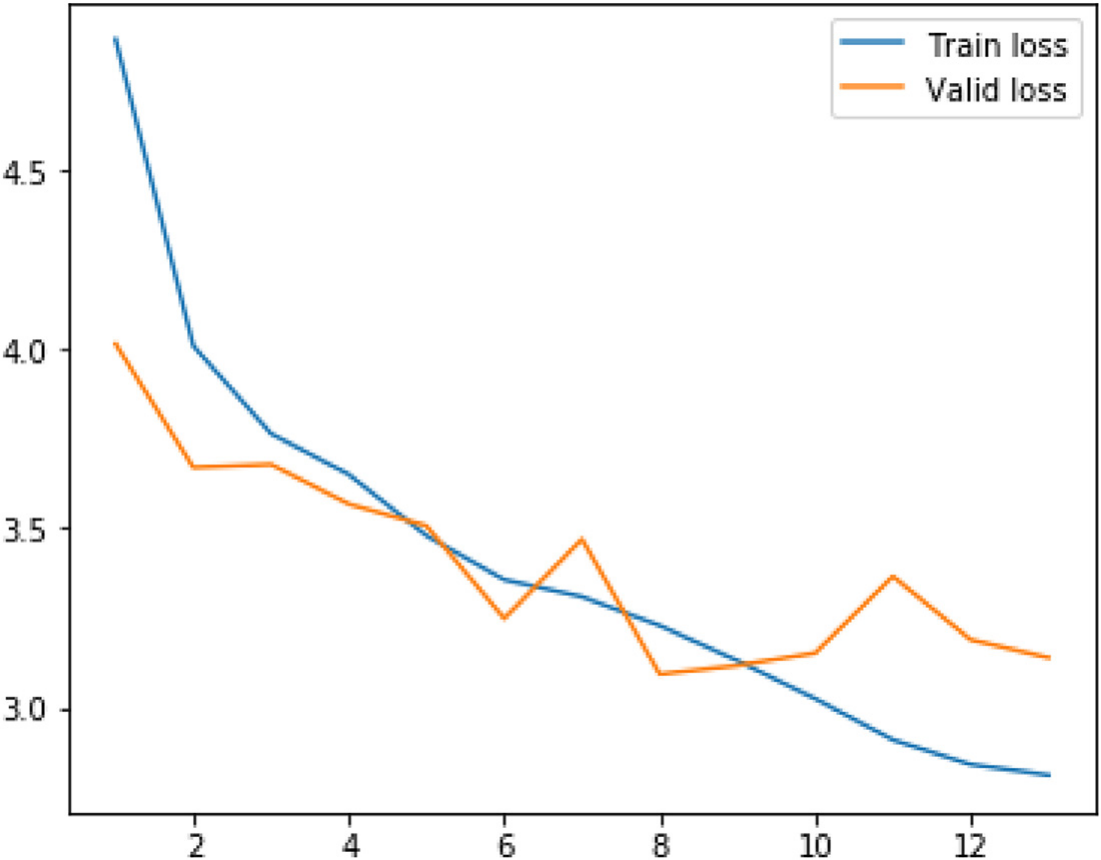
Plot for loss and validation loss against number of epochs

The plot shows the variation of loss and validation loss with respect to the number of training epochs.

**Fig. 7:**
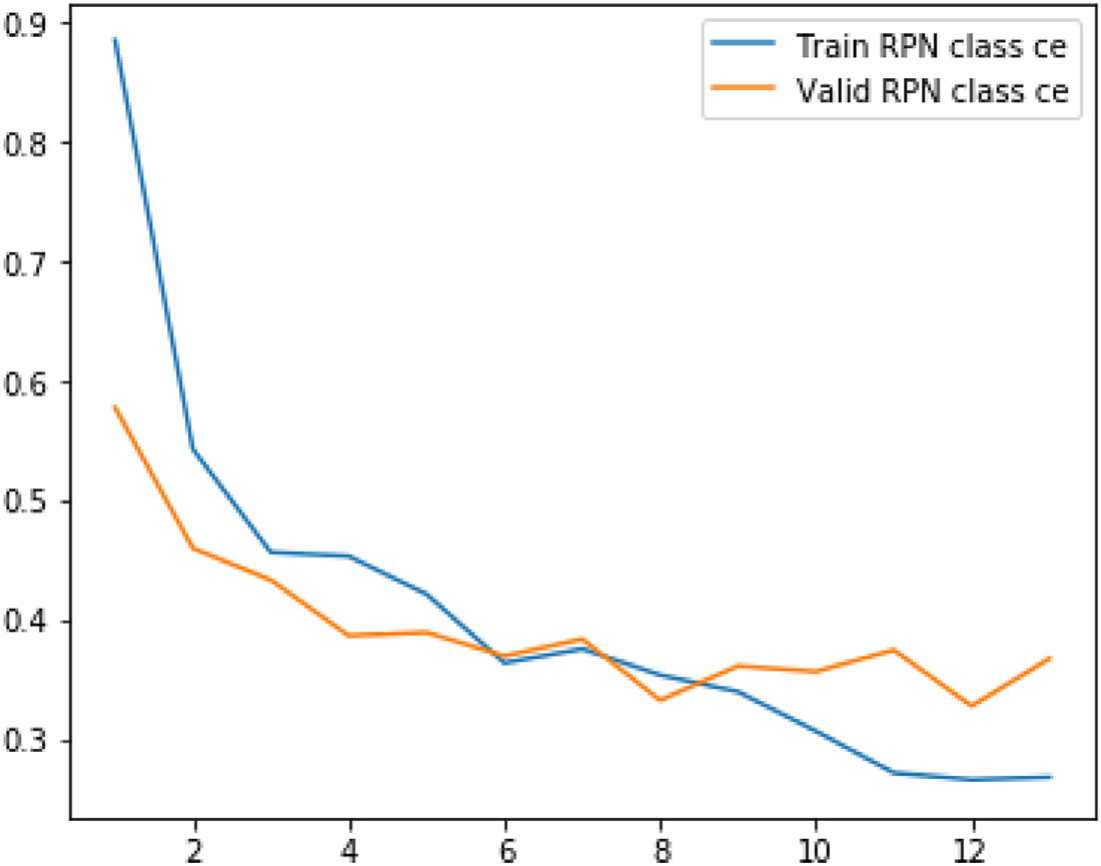
Plot for RPN Class loss and validation RPN Class loss against number of epochs.

**Fig. 8:**
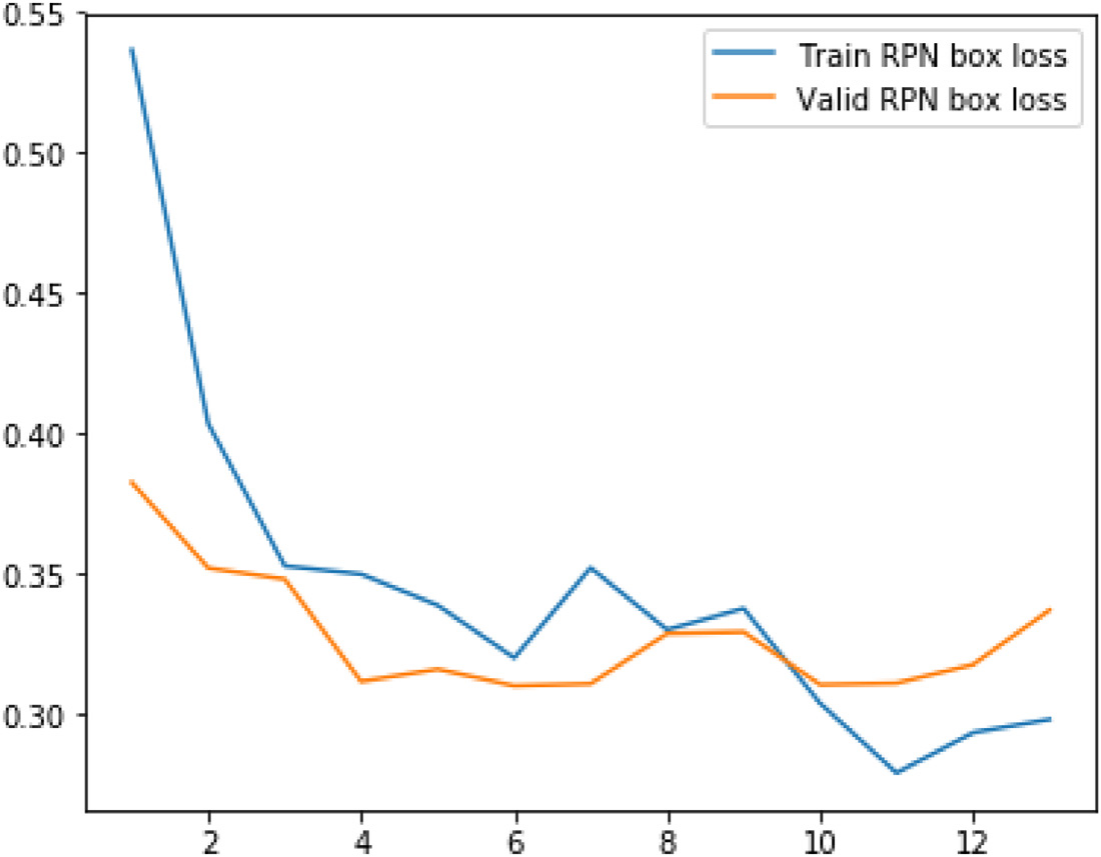
Plot for RPN box loss and validation RPN box loss against number of epochs.

**Fig. 9:**
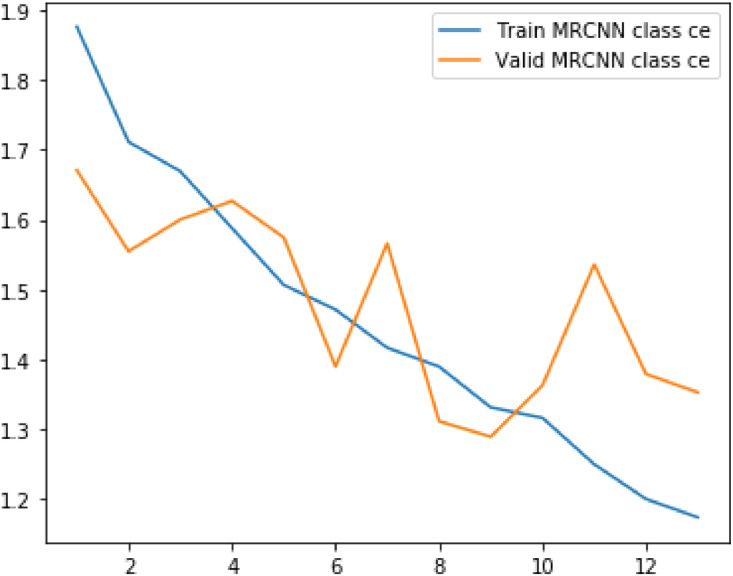
Plot for MRCNN class loss and validation MRCNN class loss against number of epochs.

**Fig. 10:**
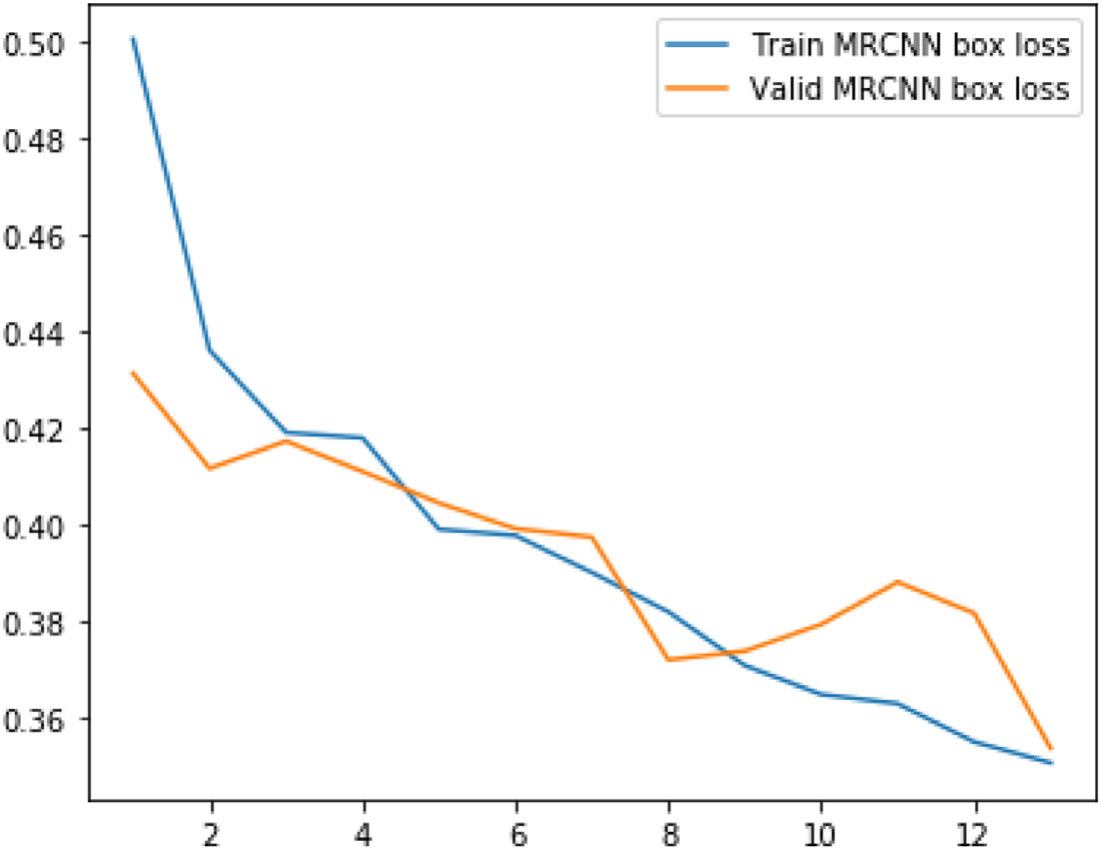
Plot for MRCNN box loss and validation MRCNN box loss against number of epochs.

**Fig. 11:**
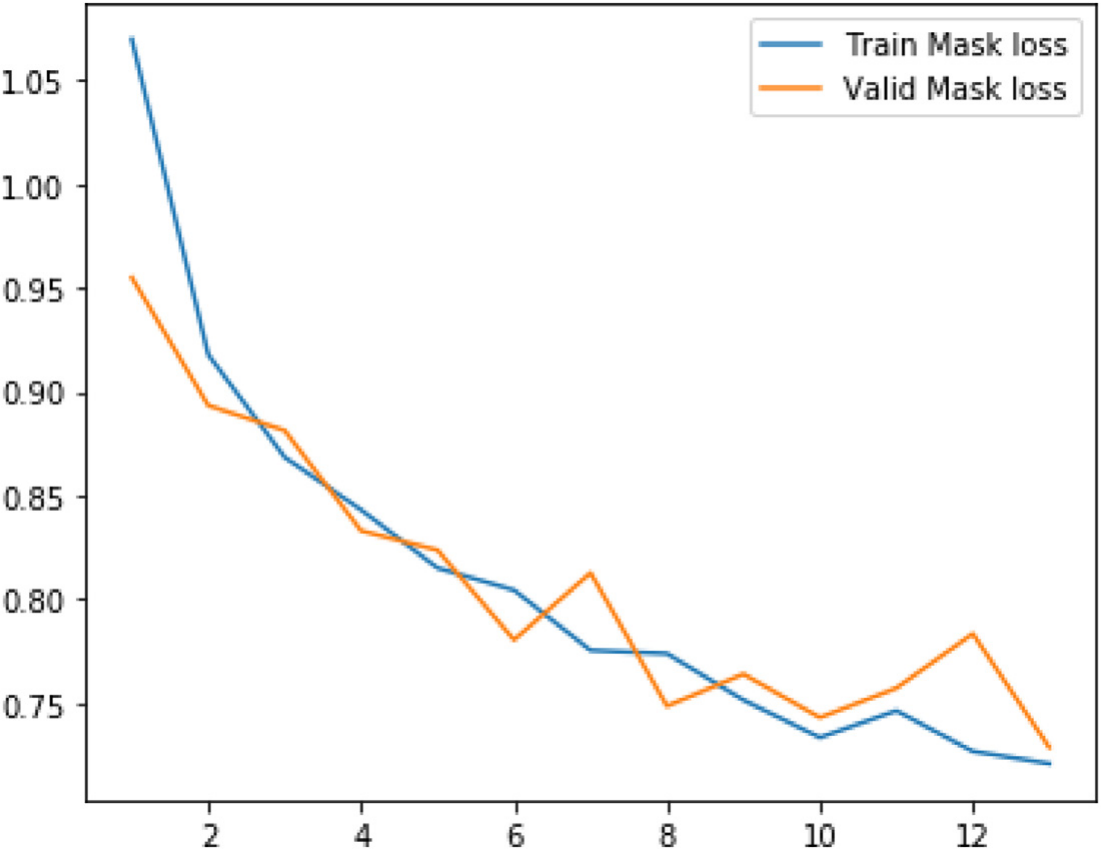
Plot for mask loss and validation mask loss against number of epochs.

## Conclusion

In this paper, a novel approach for pneumothorax segmentation using chest X-rays based on the Mask R-CNN model and medical transfer learning is proposed. The proposed approach demonstrated state- of-the-art performance on the task of pneumothorax segmentation, outperforming existing methods in terms of segmentation accuracy and speed.

One of the main strengths of proposed approach is the use of the Mask R-CNN model for object detection and segmentation. This model has proven to be very effective on various computer vision tasks and has been adapted to medical image analysis with promising results. In proposed work, the Mask R-CNN model to the specific problem of pneumothorax segmentation and demonstrated its effectiveness is applied.

The proposed research also leveraged medical transfer learning techniques to further improve the performance of proposed model on this specific medical imaging task. Medical transfer learning is a promising approach for medical image analysis, as it allows models to leverage knowledge learned from other medical imaging tasks to improve performance on a specific task. In proposed work, fine-tuned the Mask R-CNN model on a large dataset of chest X-rays with pneumothorax annotations is used, and applied transfer learning techniques to further improve its performance.

The proposed approach has the potential to improve the accuracy and efficiency of pneumothorax diagnosis and treatment, which is critical for patient outcomes. By accurately identifying and localizing pneumothorax on chest X-rays, clinicians can make more informed decisions about treatment and avoid serious complications.

Overall, it is believed that proposed work demonstrates the effectiveness of the Mask R-CNN model and medical transfer learning for pneumothorax segmentation using chest X-rays and has the potential to impact clinical practice and patient outcomes.

## Future works

As with any study conducted, there is always room for improvements and further advancements. Future work in this area could explore the use of other deep learning models and transfer learning techniques for pneumothorax segmentation and other medical imaging tasks. In addition, more research is needed to evaluate the generalizability of proposed approach to other datasets and clinical settings. In this context, the accuracy achieved using neural networks can be further improved by incorporating the use of transfer learning models in a better way and use of deep learning techniques to include further detailed classification possibilities such as segmenting the regions with sub tumoral growth that include complete, core and enhanced tumor from the images and this can be further extended to achieve even better accuracy and usability of machine learning in the field of prediction of medical conditions using imagery.

## Ethics statements

Not applicable.

## CRediT authorship contribution statement

**Shwetambari Chiwhane:** Project administration, Supervision, Writing – original draft, Methodology. **Lalit Shrotriya:** Data curation, Methodology, Software, Writing – original draft, Methodology. **Amol Dhumane:** Funding acquisition, Writing – review & editing, Validation. **Sonali Kothari:** Supervision, Writing – original draft, Conceptualization. **Deepak Dharrao:** Writing – review & editing, Validation. **Pooja Bagane:** Writing – review & editing, Validation, Supervision.

## Declaration of competing interest

The authors declare that they have no known competing financial interests or personal relationships that could have appeared to influence the work reported in this paper.

## Data Availability

Data will be made available on request.
